# Efficacy, tolerability, and safety of lurasidone for acute schizophrenia: A systematic review and network meta‐analysis of phase 3 trials in Japan

**DOI:** 10.1002/npr2.12131

**Published:** 2020-08-07

**Authors:** Taro Kishi, Tadashi Nosaka, Kenji Sakuma, Makoto Okuya, Nakao Iwata

**Affiliations:** ^1^ Department of Psychiatry Fujita Health University School of Medicine Toyoake Japan; ^2^ Medical Affairs Sumitomo Dainippon Pharma Co., Ltd. Chuo‐ku Japan

**Keywords:** acute schizophrenia, efficacy/safety/tolerability, Japan clinical trial, systematic review and network meta‐analysis

## Abstract

**Introduction:**

Considering that the efficacy results of the Japan lurasidone phase 3 trials for acute schizophrenia were inconsistent, we conducted a systematic review and a random‐effect model network meta‐analysis of those trials to examine whether lurasidone was beneficial for the treatment of Japanese patients with acute schizophrenia.

**Methods:**

The study included the double‐blind, randomized trial in Japan that included patients with acute schizophrenia. Efficacy outcomes were improvement of the Positive and Negative Syndrome Scale total score (PANSS‐T, primary), positive (PANSS‐P), negative (PANSS‐N), and general (PANSS‐G) subscale scores; and Clinical Global Impression‐Severity Scale (CGI‐S) score and response rate. Other outcomes were discontinuation rates and incidence of individual adverse events.

**Results:**

We included four studies (n = 1,608). Although both lurasidone 40 mg/d (LUR40) and 80 mg/d (LUR80) outperformed placebo in PANSS‐T [standardized mean difference (95% credible interval): LUR40 = −0.298 (−0.420, −0.176), LUR80 = −0.170 (−0.320, −0.019)], PANSS‐P, and CGI‐S scores, LUR40 but not LUR80 outperformed placebo in PANSS‐N and PANSS‐G scores and response rate. LUR40 outperformed LUR80 regarding PANSS‐G score. Both LUR40 and LUR80 were associated with a higher incidence of akathisia, somnolence, and increased body weight compared with placebo. Compared with placebo, LUR40 was associated with a higher incidence of weight gain (≥7%), and LUR80 was associated with a higher incidence of dystonia and weight loss (≥7%) and higher Drug‐Induced Extrapyramidal Symptoms Scale score.

**Conclusions:**

Both LUR40 and LUR80 improved overall symptoms in Japanese patients with acute schizophrenia. However, LUR80 seemed to have a risk of extrapyramidal symptoms.

## Abstract 2

Our network meta‐analysis included four studies (n = 1,608).Both lurasidone 40 mg/d and 80 mg/d outperformed placebo in PANSS total score.

## Graphical abstract

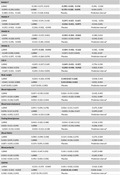

## 1 INTRODUCTION

Lurasidone (LUR) was approved for the treatment of schizophrenia and bipolar depression in Japan on March 25, 2020. Three double‐blind, randomized, placebo‐controlled, phase 3 trials (DBRPCP3T) of LUR for acute schizophrenia have been conducted.^1^, ^2^, ^3^ Two DBRPCP3T included LUR 40 mg/d (LUR40), LUR 80 mg/d (LUR80), and placebo arms.^1^, ^2^ These two DBRPCP3T showed that both LUR40 and LUR80 were not superior to placebo in the primary efficacy outcome [Positive and Negative Syndrome Scale total score (PANSS‐T)].^1^, ^2^, ^4^ Most recent DBRPCP3T showed that LUR40 outperformed placebo regarding the improvement of PANSS‐T.^3^ Thus, the efficacy results among three Japanese DBRPCP3Ts were inconsistent (Table 1); therefore, we performed a systematic review and network meta‐analysis to examine the true benefits and efficacy of LUR40 and LUR80 in Japanese patients with acute schizophrenia.

**Table 1:** Study characteristics

Study and study duration	Patients characteristics	Total n (%Japanese)	Intervention (n)	Mean age/%female	PANSS‐T at baseline	Efficacy outcomes (primary analysis)^a^
P2‐J001^7^, 8 w DBRT	SZ (IP or OP, ICD‐10) with the following symptoms: delusion, hallucination, lack of spontaneity, neurotic condition or depression	208 (100%)	LUR80 (65)	42.4 ± 11.7/45.6%	85.5 ± 21.4	Not comparisons among the treatment groups (FAS, LOCF)^b^
			LUR40 (72)		81.7 ± 18.9	
			LUR20 (71)		83.3 ± 19.6	
Higuchi 2019^1^, P3‐J056 (NCT01614899), 6 w DBRPCT [receding 3‐7 d placebo period, PLA responder were excluded (≥ 20% reduction in the PANSS‐T for the phase)]	SZ (IP, DSM‐IV‐TR) with acute exacerbation (< 2 m). PANSS‐T ≥ 80, including a score of ≥ 4 on two or more of the following PANSS items: delusions (P1), conceptual disorganization (P2), hallucinations (P3), suspiciousness (P6), and unusual thought content (G9) at screening and baseline visits.	457 (43.3%)	LUR80 (155)	43.6 ± 13.8/47.4%	101.0 ± 15.9	PANSS‐T: LUR80 = PLA PANSS‐P: LUR80 > PLA PANSS‐N: LUR80 = PLA PANSS‐G: LUR80 = PLA PANSS‐T: LUR40 = PLA PANSS‐P: LUR40 = PLA PANSS‐N: LUR40 = PLA PANSS‐G: LUR40 = PLA (mITT, MMRM)
			LUR40 (150)	42.1 ± 13.0/45.3%	102.8 ± 16.3	
			PLA (152)	42.6 ± 13.4/41.1%	101.5 ± 14.1	
Higuchi 2019^2^, P3‐J002 (NCT00711269), 6 w DBRPCT [receding 3‐7 d placebo period, PLA responder were excluded (≥ 20% reduction in the PANSS‐T for the phase)]	SZ (IP, DSM‐IV) with acute exacerbation. PANSS‐T ≥ 70, including a score of ≥ 4 on one or more of the seven positive symptom subscale items in the PANSS at screening and baseline visits.	460 (49.5%)	LUR80 (131)	45.7 ± 13.51/36.4%	92.2 ± 15.3	PANSS‐T: LUR80 = PLA PANSS‐P: LUR80 = PLA PANSS‐N: LUR80 = PLA PANSS‐G: LUR80 = PLA PANSS‐T: LUR40 = PLA PANSS‐P: LUR40 = PLA PANSS‐N: LUR40 = PLA PANSS‐G: LUR40 = PLA (FAS, LOCF)
			LUR40 (131)	45.6 ± 14.48/40.0%	91.9 ± 16.8	
			RIS4 (65)	44.8 ± 12.59/45.3%	88.9 ± 13.7	
			PLA (133)	46.0 ± 12.85/44.2%	92.8 ± 14.7	
P3‐J066^3^ (EU‐CTR 2016‐000 060‐42), 6 w DBRPCT [receding 3‐7 d placebo period, PLA responder were excluded (≥ 20% reduction in the PANSS‐T for the phase)]	SZ (IP, DSM‐IV‐TR) with acute exacerbation (< 2 m). PANSS‐T ≥ 80, including a score of ≥ 4 on two or more of the following PANSS items: delusions (P1), conceptual disorganization (P2), hallucinations (P3), suspiciousness (P6), and unusual thought content (G9) and CGI‐S ≥ 4 at screening and baseline visits.	483 (22.2%)	LUR40 (247)	41.0 ± 11.0/51.4%	102.8 ± 11.04	PANSS‐T: LUR40 > PLA PANSS‐P: LUR40 > PLA PANSS‐N: LUR40 > PLA PANSS‐G: LUR40 > PLA (ITT, MMRM)
			PLA (236)	39.3 ± 11.44/48.5%	101.7 ± 11.45	

Note

ITT: defined all subjects who were randomized at baseline, received at least 1 dose of double‐blind study drug, and had both baseline and at least one postbaseline assessment of PANSS total score.

mITT: the same as the ITT population but excluded data from PANSS assessments that were performed within 12 hours after the use of lorazepam or hypnotic drugs.

LOCF: the most recently observed outcome measure is assumed to hold for all subsequent outcome assessment times.

MMRM: data collected from all patients (those who drop out as well as those who complete the study) are used to predict mean longitudinal outcomes for the treatment group.

FAS: In P2‐J001, defined subjects who were randomized and received at least one dose of study medication, excluded subjects who were not discontinued prior other antipsychotics, carbamazepine, sodium valproate and lithium carbonate, and had not at least one BPRS or PANSS assessments during the treatment period. In P3‐J002, defined subjects who were randomized and received at least one dose of study medication during the treatment period, and received PANSS assessments at baseline and at least once postbaseline during the treatment period.

BPRS: Brief Psychiatric Rating Scale, CGI‐S: Clinical Global Impression‐Severity of Illness, DBRPCT: double‐blind, randomized, placebo‐controlled trial, DBRT: double‐blind, randomized trial, DSM‐IV: Diagnostic and Statistical Manual of Mental Disorders, Fourth Edition, DSM‐IV‐TR: Diagnostic and Statistical Manual of Mental Disorders, Fourth Edition, Text Revision, FAS: full analysis set, IP: inpatient, ITT: intent‐to‐treat, LOCF: last observation carried forward, LUR: lurasidone, m: month, mITT; modified intent‐to‐treat, MMRM: mixed model for repeated measurements, OP; outpatient, P2: phase II trial, P3: phase III trial, PANSS: Positive and Negative Syndrome Scale (‐T; total score, ‐P; positive subscale score, ‐N; negative subscale score, ‐G; general psychopathology subscale score), PLA: placebo, RIS: risperidone, SZ: schizophrenia

^a^ Primary outcome was underlined.

^b^ The primary outcomes were change from baseline in BPRS and PANSS total score (as a dose‐response evaluation by the maximum contrast method)

## 2 METHODS

This study was performed according to the Preferred Reporting Items for Systematic Reviews and Meta‐Analyses guidelines (Appendix S1).^5^ The literature search, data extraction, and data input into spreadsheets for analysis were done simultaneously and independently by at least two authors (TK, TN, KS, and MO). The authors double‐checked the data transfer accuracy and calculations in the study.

### 2.1 Literature search

We included only double‐blind randomized trials. We used Embase, PubMed, Scopus, and Cochrane Library; without language restrictions, from the date of inception of these databases to June 25, 2020. The following search strategy keywords were used: (lurasidone) AND (schizophrenia) AND (Asian OR Japan). Additional searches were conducted on ClinicalTrials.gov.^6^ We also performed a hand search to identify any other articles. Ultimately, three DBRPCP3Ts^1^, ^2^, ^3^ and one double‐blind, randomized, phase 2 trial (DBRP2T)^7^ met the criteria and were included in the present systematic review.

### 2.2 Data extraction and data synthesis

Intention‐to‐treat or modified intention‐to‐treat (full analysis set) data were used in the analysis.^8^, ^9^ We included outcomes that reported data from at least three selected trials. Efficacy outcomes were improvement of PANSS‐T (primary for efficacy), PANSS positive (PANSS‐P), negative (PANSS‐N), and general (PANSS‐G) subscale scores, Clinical Global Impression‐Severity Scale (CGI‐S)^10^ score, and response rate (≥20% reduction in the total PANSS‐T score). Other outcomes were all‐cause discontinuation, discontinuation due to adverse events, and incidence of individual adverse events. The methodological qualities of the included articles were assessed according to the Cochrane risk‐of‐bias tool.^9^

### 2.3 Meta‐analysis methods

A Bayesian network meta‐analysis based on random‐effects models^11^ was conducted using the netmeta package.^12^ Standardized mean differences (SMDs), risk ratios, and their 95% credible intervals (95%Crls) were calculated for continuous and dichotomous data, respectively. For cases where the risk ratios showed statistically significant between‐group differences with respect to treatment efficacy, discontinuation rates or the incidence of individual adverse events based on RRs were significant; either the number needed to treat for benefit (NNTB) or for harm (NNTH) was calculated from the risk difference (RD), using the formula NNTB/NNTH = 1/RD. We assessed network heterogeneity through τ^2^ and I^2^ statistic using the netmeta.^12^ We inferred the magnitude of heterogeneity by comparing the estimated τ^2^ to empirical distributions of heterogeneity typically found in meta‐analyses.^13^ Due to the fact that we found local heterogeneity between LUR80 and placebo for the primary outcome, we performed the following two sensitivity analysis: (a) first sensitivity analysis excluding DBRP2T because this study did not have a placebo washout period before randomization and (b) second sensitivity analysis excluding patients who received antipsychotics, with dosage of ≥12 mg/d of haloperidol equivalent before randomization. We did a statistical evaluation of the consistency using the design‐by‐treatment test (globally) and by the node‐splitting approach or Separate Direct from Indirect Evidence test (locally). We incorporated the results into the Confidence in Network Meta‐Analysis (CINeMA) application to assess the credibility of findings from the network meta‐analysis.^14^ We did not explore publication bias because only four studies included in our study. We also conducted a primary single‐group summary meta‐analysis to calculate the mean improvement PANSS‐T score, mean all‐cause discontinuation rate, and 95% confidence intervals (95%CIs) in LUR40, LUR80, and placebo groups using Comprehensive Meta‐Analysis Software Version 3.^15^

## 3 RESULTS

### 3.1 Study characteristics

The result of the literature search is shown in Figure S1. The search identified three DBRPCP3Ts^1^, ^2^, ^3^ and one DBRP2T^7^(n = 1,608). All the studies were sponsored by pharmaceutical companies. The three DBRPCP3Ts were international studies and two studies were unpublished.^3^, ^7^ Although DBRP2T did not have a severity threshold of schizophrenia at baseline, because PANSS‐T scores were more than 80, we included DBRP2T as an acute schizophrenia trial. The methodological quality of all the studies was high as assessed with the Cochrane risk‐of‐bias tool (Table S1). The study and patient characteristics at baseline are presented in Table 1. Although the study duration of the DBRPCP3Ts was 6 weeks, that of DBRP2T were 8 weeks. DBRP2T did not have a placebo arm. The results of the meta‐analysis are shown in Tables 2 and 3; Appendix S2.

**Table 2:** Results of the network meta‐analysis: continuous variable

PANSS‐T			
LUR40	−0.128 (−0.271, 0.015)	−**0.298 (−0.420, −0.176)**	−0.496, −0.100
−0.099 (−0.250, 0.051)	LUR80	−**0.170 (−0.320, −0.019)**	−0.414, 0.075
−0.288 (−0.412, −0.165)	−0.151 (−0.318, 0.016)	Placebo	Prediction interval*

PANSS‐P			
LUR40	−0.040 (−0.191, 0.110)	−**0.297 (−0.427, −0.167)**	−0.542, −0.051
−0.008 (−0.166, 0.149)	LUR80	−**0.256 (−0.415, −0.097)**	−0.543, 0.031
−0.291 (−0.423, −0.160)	−0.226 (−0.402, −0.049)	Placebo	Prediction interval*

PANSS‐N			
LUR40	−0.082 (−0.225, 0.061)	−**0.183 (−0.304, −0.062)**	−0.380, 0.014
−0.078 (−0.228, 0.073)	LUR80	−0.101 (−0.252, 0.049)	−0.345, 0.143
−0.173 (−0.296, −0.050)	−0.114 (−0.281, 0.053)	Placebo	Prediction interval*

PANSS‐G			
LUR40	−**0.177 (−0.320, −0.034)**	−**0.284 (−0.406, −0.162)**	−0.482, −0.086
−0.149(−0.300, 0.002)	LUR80	−0.107 (−0.257, 0.043)	−0.351, 0.137
−0.273 (−0.397, −0.150)	−0.092 (−0.260, 0.074)	Placebo	Prediction interval*

CGI‐S			
LUR40	−0.029 (−0.227, 0.169)	−**0.269 (−0.431, −0.107)**	−0.796, 0.258
0.006 (−0.203, 0.215)	LUR80	−**0.240 (−0.438, −0.042)**	−0.823, 0.343
−0.269 (−0.431, −0.107)	−0.205 (−0.414, 0.005)	Placebo	Prediction interval*

Body weight			
LUR40	−0.064 (−0.206, 0.078)	**0.148 (0.027, 0.268)**	−0.048, 0.343
−0.061 (−0.210, 0.088)	LUR80	**0.211 (0.062, 0.361)**	−0.031, 0.454
0.147 (0.025, 0.269)	0.217 (0.051, 0.383)	Placebo	Prediction interval*

Blood triglyceride			
LUR40	0.037 (−0.158, 0.232)	0.026 (−0.154, 0.206)	−0.442, 0.493
0.077 (−0.125, 0.280)	LUR80	−0.011 (−0.222, 0.200)	−0.511, 0.489
0.046 (−0.138, 0.229)	0.010 (−0.223, 0.244)	Placebo	Prediction interval*

Blood total cholesterol			
LUR40	0.091 (−0.096, 0.278)	0.056 (−0.115, 0.227)	−0.375, 0.487
0.078 (−0.117, 0.272)	LUR80	−0.035 (−0.236, 0.167)	−0.499, 0.429
0.082 (−0.092, 0.257)	−0.096 (−0.319, 0.128)	Placebo	Prediction interval*

Fasting blood glucose			
LUR40	0.043 (−0.102, 0.188)	−0.014 (−0.140, 0.112)	−0.218, 0.190
0.042 (−0.111, 0.194)	LUR80	−0.057 (−0.210, 0.096)	−0.306, 0.192
−0.024 (−0.152, 0.103)	−0.042 (−0.212, 0.128)	Placebo	Prediction interval*

Blood HbA1c			
LUR40	0.080 (−0.098, 0.258)	0.114 (−0.046, 0.275)	−0.275, 0.504
0.110 (−0.075, 0.295)	LUR80	0.034 (−0.156, 0.225)	−0.389, 0.458
0.087 (−0.076, 0.250)	0.119 (−0.091, 0.330)	Placebo	Prediction interval*

Blood prolactin			
LUR40	0.023 (−0.147, 0.192)	0.141 (−0.010, 0.292)	−0.207, 0.489
0.053 (−0.124, 0.230)	LUR80	0.119 (−0.062, 0.299)	−0.265, 0.502
0.118 (−0.035, 0.272)	0.196 (−0.003, 0.396)	Placebo	Prediction interval*

DIEPSS			
LUR40	−0.221 (−0.374, −0.069)	0.101 (−0.032, 0.233)	−0.163, 0.365
−0.183 (−0.343, −0.023)	LUR80	**0.322 (0.161, 0.484)**	0.018, 0.626
0.108 (−0.026, 0.243)	0.357 (0.178, 0.537)	Placebo	Prediction interval*

Note

Standardized mean difference (95% credible interval).

Results from pair‐wise meta‐analysis are presented in the left lower half and results from network meta‐analysis in the upper right half.

The bold‐face value indicated the statistically significant.

CGI‐S, Clinical Global Impressions‐severity of illness; DIEPSS, Drug‐Induced Extrapyramidal Symptoms Scale; LUR, lurasidone; PANSS, Positive and Negative Syndrome Scale score (‐T; total, ‐P; positive subscale, ‐N; negative subscale, ‐G; general psychopathology subscale).

* Prediction interval when comparing active drug with placebo.

### 3.2 Efficacy

Although both LUR40 and LUR80 outperformed placebo in PANSS‐T [SMD (95%Crl): LUR40 = −0.298 (−0.420, −0.176), LUR80 = −0.170 (−0.320, −0.019)], PANSS‐P, and CGI‐S scores, LUR40 but not LUR80 outperformed placebo in PANSS‐N and PANSS‐G scores and response rate. LUR40 outperformed LUR80 regarding PANSS‐G score. Mean change PANSS‐T and 95%CI in LUR40, LUR80, and placebo were −14.51 (−22.25, −6.78), −8.57 (−17.14, 0.208), and −9.49 (−15.88, −3.09).

### 3.3 Safety, tolerability, and adverse effects

Both lurasidone 40 mg/d (LUR40) and 80 mg/d (LUR80) were associated with a higher incidence of akathisia, somnolence, and increased body weight gain compared with placebo. LUR40 was associated with a higher incidence of weight gain (≥7%) compared with placebo. LUR80 was associated with a higher incidence of dystonia and weight loss (≥7%) and having higher Drug‐Induced Extrapyramidal Symptoms Scale score compared with placebo. However, the risk of LUR40 and LUR80 for these outcomes was small. Only LUR40 was associated with a lower incidence of schizophrenia as an adverse event compared with placebo. There were not significant differences in other tolerability and safety outcomes among the groups. Mean all‐cause discontinuation and 95%CIs in LUR40, LUR80, and placebo were 27.6% (21.1%, 35.2%), 34.6% (24.6%, 46.2%), and 29.8% (23.2%, 37.4%).

### 3.4 Heterogeneity, inconsistency, and the evidence for the network meta‐analysis graded by the cinema system

Although we did not detect global heterogeneity or global consistency for the primary outcome, we found local heterogeneity between LUR80 and placebo (Appendix S2). We performed two sensitivity analyses (detailed information in the Methods section). Both the first and second sensitivity analyses demonstrated that LUR80 outperformed placebo [SMD (95% Crl): first sensitivity analysis = −0.191 (−0.348, −0.034) and second sensitivity analysis = −0.182 (−0.367, −0.003)]. However, CINeMA judged the major concern for heterogeneity in both sensitivity analyses. The confidence in the evidence for LUR40 and LUR80 in the primary outcome was moderate and low, respectively. We did not assess publication bias because the number of studies was small. Therefore, the confidence in the evidence for most of the outcomes was usually low or very low (Appendix S2).

## 4 DISCUSSION

Our meta‐analysis demonstrated that both LUR40 and LUR80 lead to a significant benefit for acute schizophrenia treatment in Japanese patients. The failure of these two DBRPCP3Ts^1^, ^2^ might be due to a type II error (ie, low statistical power). Another reasons might be that the placebo response of LUR’s DBRPCP3Ts was large compared with those of other antipsychotic's DBRPCP3Ts.^16^ Although there was no significant difference in the primary outcome between LUR40 and LUR80, the effect size of LUR40 seems to be larger than that of LUR80. Dose‐response meta‐analysis of antipsychotic drugs for acute schizophrenia showed that the 95% effective dose of lurasidone was 147 mg/d.^17^ Further studies are needed to investigate why LUR’s efficacy did not increase dose dependently in Japanese patients (eg, pharmacogenetics on drug response and drug metabolism).

Although both LUR40 and LUR80 increased the mean body weight with very small effect on size, the incidence of weight loss seemed to be larger that of weight gain in all groups [incidence of weight gain (≥7%) and weight loss (≥7%) in all groups were as follows; LUR40 = 3.8% (2.4%, 5.9%) and 4.9% (2.0%, 11.7%), LUR80 = 1.4% (0.5%, 3.7%) and 4.3% (2.4% and 7.3%), and placebo = 1.5% (0.7% and 3.1%) and 5.9% (2.4%, 13.6%)]. Both LUR40 and LUR80 did not also influence the blood examination related to glucose and lipid metabolism. Thus, LUR seems to have very little effect on body weight and glucose and lipid metabolism, similar to the results of previous meta‐analysis.^18^ On the other hand, LUR80 seemed to have a risk of extrapyramidal symptoms compare with LUR40, although there were no significant differences in the outcomes between the groups.

There were several limitations to this study. First, because the DBRPCP3Ts included Japanese and non‐Japanese patients, the results may not directly reflect the clinical practice in Japan. However, the number of Japanese patients in these trials was small. Second, although concomitant medication might influence the results in each trial, there were no significant differences in these outcomes among the groups.

In conclusion, both LUR40 and LUR80 improved the overall symptoms of acute schizophrenia in Japanese patients. However, LUR80 seemed to be associated with a risk of extrapyramidal symptoms compare with LUR40.

## CONFLICT OF INTEREST

The present study was supported by the Grant‐in‐Aid for Scientific Research (C) (19K08082). Mr Nosaka is full‐time employee of Sumitomo Dainippon Pharma Co., Ltd. Other authors have declared that there are no conflicts of interest relating to the subject of this study.

Dr Kishi received speaker's honoraria from Daiichi Sankyo, Dainippon Sumitomo, Eisai, Janssen, Otsuka, Meiji, Mochida, MSD, and Tanabe‐Mitsubishi (Yoshitomi); as well as research grants from the Japanese Ministry of Health, Labour and Welfare, Grant‐in‐Aid for Scientific Research (C), and Fujita Health University School of Medicine.

Dr Sakuma has received speaker's honoraria from Eisai, Kissei, Meiji, Otsuka, and Torii; and has received a Fujita Health University School of Medicine research grant, as well as a Grant‐in‐Aid for Young Scientists (B).

Dr Okuya has received speaker's honoraria from Meiji.

Dr Iwata received speaker's honoraria from Astellas, Dainippon Sumitomo, Eli Lilly, GlaxoSmithKline, Janssen, Yoshitomi, Otsuka, Meiji, Shionogi, Novartis, and Pfizer; along with research grants from GlaxoSmithKline, Meiji, and Otsuka.

Data Repository: The data that support the findings of this study are openly available in the articles of three studies^1^, ^2^, ^3^, ^7^ that cited in this paper.

Approval of the research protocol by an Institutional Reviewer Board: Not applicable.

Informed Consent: Not applicable.

Registry and the Registration No. of the study/trial: Not applicable.

Animal Studies: Not applicable.

## AUTHOR CONTRIBUTIONS

TK involved in the study conception and design and performed the statistical analysis. TK, TN, KS, and MO performed the acquisition and interpretation of the data. All the authors wrote the manuscript. NI supervised the review.

5

**Table 3:** Results of the network meta‐analysis: dichotomous variable

Response rate (≥ 20% reduction in PANSS‐T)			
LUR40	0.918 (0.774, 1.088)	**0.826 (0.716, 0.953)** *, ^a^	0.518, 1.318
0.963 (0.805, 1.151)	LUR80	0.900 (0.763, 1.062)	0.545, 1.486
0.826 (0.716, 0.953)	0.937 (0.789, 1.113)	Placebo	Prediction interval*

All‐cause discontinuation			
LUR40	0.905 (0.732, 1.119)	0.869 (0.713, 1.058)	0.613, 1.230
0.927 (0.744, 1.156)	LUR80	0.960 (0.765, 1.204)	0.648, 1.421
0.892 (0.730, 1.091)	0.947 (0.739, 1.214)	Placebo	Prediction interval*

Discontinuation due to adverse events			
LUR40	0.871 (0.488, 1.556)	0.879 (0.518, 1.493)	0.295, 2.623
0.868 (0.478, 1.577)	LUR80	1.010 (0.519, 1.962)	0.283, 3.605
0.991 (0.576, 1.706)	0.748 (0.341, 1.644)	Placebo	Prediction interval*

At least one adverse event			
LUR40	0.989 (0.916, 1.067)	1.031 (0.948, 1.121)	0.899, 1.182
0.999 (0.924, 1.079)	LUR80	1.042 (0.951, 1.143)	0.898, 1.210
1.039 (0.953, 1.133)	1.049 (0.949, 1.160)	Placebo	Prediction interval*

Akathisia			
LUR40	0.684 (0.441, 1.062)	**2.052 (1.139, 3.699)** ^b^	0.788, 5.342
0.676 (0.431, 1.058)	LUR80	**2.999 (1.639, 5.488)** ^c^	1.124, 8.000
2.131 (1.169, 3.884)	2.791 (1.430, 5.446)	Placebo	Prediction interval*

Anxiety			
LUR40	0.899 (0.286, 2.826)	0.600 (0.241, 1.494)	0.027, 13.328
0.883 (0.253, 3.081)	LUR80	0.668 (0.225, 1.985)	0.023, 19.321
0.631 (0.251, 1.588)	0.768 (0.244, 2.421)	Placebo	Prediction interval*

Constipation			
LUR40	1.292 (0.680, 2.456)	0.915 (0.531, 1.575)	0.315, 2.660
1.259 (0.651, 2.435)	LUR80	0.708 (0.373, 1.342)	0.213, 2.351
0.946 (0.547, 1.636)	0.618 (0.319, 1.199)	Placebo	Prediction interval*

Diarrhea			
LUR40	1.603 (0.680, 3.782)	0.757 (0.386, 1.484)	0.254, 2.259
1.601 (0.668, 3.837)	LUR80	0.472 (0.197, 1.134)	0.114, 1.958
0.792 (0.399, 1.570)	0.428 (0.167, 1.097)	Placebo	Prediction interval*

Dizziness			
LUR40	1.045 (0.357, 3.056)	1.941 (0.737, 5.111)	0.403, 9.349
0.997 (0.325, 3.058)	LUR80	1.857 (0.548, 6.292)	0.256, 13.468
1.962 (0.739, 5.208)	1.658 (0.399, 6.885)	Placebo	Prediction interval*

Dry mouth			
LUR40	0.603 (0.130, 2.805)	1.451 (0.296, 7.124)	0.110, 19.215
0.599 (0.116, 3.085)	LUR80	2.407 (0.421, 13.756)	0.142, 40.809
1.366 (0.264, 7.056)	2.063 (0.268, 15.874)	Placebo	Prediction interval*

Dystonia			
LUR40	0.499 (0.199, 1.250)	2.998 (0.724, 12.416)	0.298, 30.123
0.499 (0.196, 1.270)	LUR80	**6.013 (1.508, 23.987)** ^d^	0.636, 56.855
2.955 (0.702, 12.446)	5.964 (1.347, 26.406)	Placebo	Prediction interval*

Headache			
LUR40	1.364 (0.775, 2.402)	1.141 (0.727, 1.792)	0.549, 2.373
1.342 (0.740, 2.434)	LUR80	0.837 (0.470, 1.491)	0.327, 2.138
1.114 (0.707, 1.756)	0.783 (0.420, 1.459)	Placebo	Prediction interval*

Insomnia			
LUR40	0.791 (0.498, 1.255)	0.891 (0.561, 1.416)	0.314, 2.526
0.887 (0.548, 1.434)	LUR80	1.127 (0.669, 1.898)	0.371, 3.426
0.851 (0.529, 1.368)	1.454 (0.808, 2.615)	Placebo	Prediction interval*

Muscle rigidity			
LUR40	1.061 (0.434, 2.593)	1.444 (0.475, 4.392)	0.237, 8.789
1.075 (0.436, 2.646)	LUR80	1.361 (0.419, 4.419)	0.201, 9.212
1.589 (0.501, 5.042)	1.312 (0.352, 4.888)	Placebo	Prediction interval*

Nausea			
LUR40	1.130 (0.664, 1.925)	1.227 (0.644, 2.337)	0.367, 4.105
1.217 (0.710, 2.084)	LUR80	1.085 (0.543, 2.168)	0.303, 3.885
1.205 (0.620, 2.343)	1.281 (0.597, 2.748)	Placebo	Prediction interval*

Rash			
LUR40	0.864 (0.249, 2.993)	0.808 (0.257, 2.543)	0.126, 5.199
0.807 (0.217, 2.995)	LUR80	0.936 (0.271, 3.237)	0.125, 7.018
0.826 (0.253, 2.696)	0.960 (0.251, 3.670)	Placebo	Prediction interval*

Schizophrenia			
LUR40	0.755 (0.495, 1.150)	**0.633 (0.442, 0.907)** ^e^	0.353, 1.135
0.751 (0.479, 1.177)	LUR80	0.839 (0.572, 1.231)	0.450, 1.564
0.636 (0.442, 0.914)	0.826 (0.555, 1.230)	Placebo	Prediction interval*

Serious adverse event			
LUR40	1.137 (0.526, 2.458)	0.831 (0.416, 1.660)	0.270, 2.556
1.197 (0.546, 2.626)	LUR80	0.731 (0.336, 1.591)	0.207, 2.585
0.841 (0.418, 1.690)	0.768 (0.343, 1.718)	Placebo	Prediction interval*

Somnolence			
LUR40	1.135 (0.640, 2.010)	**4.799 (1.539, 14.969)** ^f^	0.757, 30.435
1.093 (0.615, 1.944)	LUR80	**4.230 (1.326, 13.496)** ^g^	0.643, 27.827
4.717 (1.444, 15.404)	3.307 (0.919, 11.898)	Placebo	Prediction interval*

Tremor			
LUR40	0.559 (0.289, 1.080)	0.882 (0.418, 1.865)	0.262,2.974
0.498 (0.251, 0.988)	LUR80	1.579 (0.804, 3.101)	0.528,4.724
0.883 (0.394, 1.979)	1.482 (0.725, 3.027)	Placebo	Prediction interval*

Use of sleeping pills			
LUR40	1.007 (0.896, 1.131)	1.017 (0.908, 1.139)	0.793, 1.304
1.014 (0.901, 1.140)	LUR80	1.010 (0.897, 1.137)	0.779, 1.310
1.017 (0.908, 1.139)	1.019 (0.903, 1.149)	Placebo	Prediction interval*

Use of anxiolytic			
LUR40	0.949 (0.837, 1.075)	1.030 (0.909, 1.167)	0.783, 1.354
0.969 (0.853, 1.102)	LUR80	1.086 (0.952, 1.238)	0.813, 1.449
1.031 (0.910, 1.168)	1.116 (0.974, 1.279)	Placebo	Prediction interval*

Use of Anticholinergic drugs			
LUR40	0.989 (0.672, 1.456)	1.475 (0.938, 2.318)	0.449, 4.840
0.950 (0.643, 1.404)	LUR80	1.491 (0.932, 2.386)	0.446, 4.982
1.593 (0.997, 2.547)	1.289 (0.780, 2.130)	Placebo	Prediction interval*

Vomiting			
LUR40	0.869 (0.508, 1.487)	1.241 (0.657, 2.346)	0.442, 3.489
0.886 (0.516, 1.522)	LUR80	1.428 (0.757, 2.694)	0.509, 4.004
1.291 (0.672, 2.481)	1.508 (0.781, 2.913)	Placebo	Prediction interval*

Weight gain (≥ 7%)			
LUR40	3.057 (0.985, 9.482)	**2.589 (1.065, 6.293)** ^h^	0.368, 18.198
2.684 (0.842, 8.558)	LUR80	0.847 (0.245, 2.926)	0.056, 12.874
2.532 (1.034, 6.200)	0.792 (0.214, 2.939)	Placebo	Prediction interval*

Weight loss (≥ 7%)			
LUR40	1.760 (0.892, 3.474)	0.835 (0.501, 1.391)	0.272, 2.559
1.787 (0.895, 3.567)	LUR80	**0.474 (0.245, 0.920)** ^i^	0.111, 2.031
0.836 (0.502, 1.393)	0.477 (0.244, 0.933)	Placebo	Prediction interval*

QTcF interval 450msec			
LUR40	1.145 (0.418, 3.135)	0.990 (0.411, 2.385)	0.136, 7.182
1.018 (0.359, 2.890)	LUR80	0.864 (0.319, 2.338)	0.093, 8.035
1.047 (0.431, 2.542)	0.833 (0.294, 2.356)	Placebo	Prediction interval*

Note

Risk ratio (95%CrI).

Results from pair‐wise meta‐analysis are presented in the left lower half and results from network meta‐analysis in the upper right half.

The bold‐face value indicated the statistically significant.

95%CrI: 95% credible interval, LUR: lurasidone, NNTB (NNTH): number needed to treat to benefit (harm), PANSS‐T: Positive and Negative Syndrome Scale total score.

^a^ NNTB (95%CrI): 8.9 (5.6, 21.7).

^b^ NNTH (95%CrI): 34.5 (19.2, 200.0).

^c^ NNTH (95%CrI): 14.1 (9.1, 31.2).

^d^ NNTH (95%CrI): 32.3 (19.2, 111.1).

^e^ NNTH (95%CrI): 25.6 (13.7, 200.0).

^f^ NNTH (95%CrI): 33.3 (21.7, 76.9).

^g^ NNTH (95%CrI): 43.5 (21.7, 1000.0).

^h^ NNTH (95%CrI): 47.6 (25.6, 333.3).

^i^ NNTH (95%CrI): 24.4 (13.3, 142.9).

* Prediction interval when comparing active drug with placebo.

## Supporting information

Supplementary Material

Appendix S1
