# Corrigendum

**DOI:** 10.1002/npr2.12270

**Published:** 2022-09-27

In Kishi et al.,^1^ Table 2 and Appendix S2 were published with incorrect data for blood triglyceride, blood total cholesterol, and fasting blood glucose.

This study represents collaborating research between Fujita Health University School of Medicine and Sumitomo Dainippon Pharma Co., Ltd (Sumitomo). After the publication, Sumitomo realized that they had provided the incorrect clinical trial (P3‐J066) dataset regarding blood triglyceride, blood total cholesterol, and fasting blood glucose to Fujita Health University School of Medicine. The data must include only fasting measurement data, but the original data included some non‐fasting measurement data. Therefore, Sumitomo asked Dr. Kishi of Fujita Health University School of Medicine to reanalyze these parameters.

The correct Table 2 is presented below, while Appendix S2 has been corrected in the online version.

**TABLE 2:** 

Blood triglyceride			
LUR40	0.038 (−0.153, 0.230)	0.028 (−0.148, 0.205)	−0.424, 0.480
0.078 (−0.121, 0.278)	LUR80	−0.010 (−0.216, 0.197)	−0.494, 0.475
0.049 (−0.131, 0.229)	0.011 (−0.218, 0.239)	Placebo	Prediction interval*

Blood total cholesterol			
LUR40	0.091 (−0.096, 0.275)	0.051 (−0.118, 0.221)	−0.372, 0.475
0.078 (−0.115, 0.271)	LUR80	−0.038 (−0.238, 0.162)	−0.495, 0.419
0.077 (−0.096, 0.250)	−0.096 (−0.317, 0.126)	Placebo	Prediction interval*

Fasting blood glucose			
LUR40	0.052 (−0.093, 0.198)	0.007 (−0.119, 0.133)	−0.198, 0.212
0.042 (−0.111, 0.194)	LUR80	−0.045 (−0.198, 0.108)	−0.294, 0.204
−0.003 (−0.131, 0.126)	−0.045 (−0.199, 0.108)	Placebo	Prediction interval*

Sumitomo accepts full responsibility and apologizes for these errors.

## Supporting information


Figure S1
