# Supplementary material for: Efficacy, tolerability, and safety of lurasidone for acute schizophrenia: A systematic review and network meta‐analysis of phase 3 trials in Japan
Source: Neuropsychopharmacol Rep. 2020 Aug 7;40(3):314–22. doi: 10.1002/npr2.12131 (PMC7722667; doi:10.1002/npr2.12131)
Supplement: Supplementary file 1 — Supplementary Material [file NPR2-40-314-s001.docx]

**Supplementary Figure 1. Flow diagram of literature search**

Potentially relevant articles (K = 89)

Duplicate articles (K = 50)

Unique articles identified and screened (K = 39)

Articles excluded at abstract level (K = 35)

Full text articles retrieved for detailed evaluation (K =4)

Not meet inclusion criteria (K = 2)

-Not Asian or Japan population (K =2)

Hand search through the abstract of conference and clinical trials registers

(K = 2)

Studies met the inclusion criteria (K = 4)

**Supplementary Table 1. Risk of bias summary**

|  | Random sequence generation | Allocation concealment | Blinding of participants and personnel | Blinding of outcome assessment | Incomplete outcome data addressed | Selective reporting | Other sources of bias |
| --- | --- | --- | --- | --- | --- | --- | --- |
| P2-J001^7^ | Unclear | Low | Unclear | Unclear | Low | Low | Unclear |
| Higuchi 2019^1^ | Low | Low | Low | Unclear | Low | Low | Unclear |
| Higuchi 2019^2^ | Low | Low | Low | Unclear | Low | Low | Unclear |
| P3-J066^3^ | Low | Low | Low | Unclear | Low | Low | Unclear |

**Supplementary Appendix 2. CINeMA confidence rating**

CINeMA confidence rating

(1) Within-study bias

We referred to the following article:

Furukawa, TA et al. (2016): Comparative efficacy and acceptability of first-generation and second-generation antidepressants in the acute treatment of major depression. Protocol for a network meta-analysis. In: BMJ open 6 (7), e010919. DOI: 10.1136/bmjopen-2015-010919.

(2) Across-studies bias

Funnel plots with fewer than 10 studies are not meaningful. Therefore, all comparisons were “Major concerns.”

(3) Indirectness

No indirectness assumed.

(4) Imprecision

A clinically meaningful threshold was set at a RR of higher or lower than 1 at a standardized mean difference of higher or lower than 0.

(5) Heterogeneity

Use recommendations automatically provided by CINeMA.

(6) Incoherence

Downgrade evidence only if indirect evidence or if significant inconsistency was detected.

PANSS-T

τ^2^ = 0.000, random-effects design-by-treatment interaction model: p = 0.261

|  | Number of studies | Within-study bias | Across-studies bias | Indirectness | Imprecision | Heterogeneity | Incoherence | Confidence rating |
| --- | --- | --- | --- | --- | --- | --- | --- | --- |
| LUR40 vs LUR80 | 3 | Low risk | Major concerns | No concerns | Major concerns | No concerns | No concern | Low |
| LUR40 vs Placebo | 3 | Low risk | Major concerns | No concerns | No concerns | No concerns | No concern | Moderate |
| LUR80 vs Placebo | 2 | Low risk | Major concerns | No concerns | No concerns | Major concerns | No concern | Low |

PANSS-P

τ^2^ = 0.002, random-effects design-by-treatment interaction model: p = 0.284

|  | Number of studies | Within-study bias | Across-studies bias | Indirectness | Imprecision | Heterogeneity | Incoherence | Confidence rating |
| --- | --- | --- | --- | --- | --- | --- | --- | --- |
| LUR40 vs LUR80 | 3 | Low risk | Major concerns | No concerns | Major concerns | No concerns | No concern | Low |
| LUR40 vs Placebo | 3 | Low risk | Major concerns | No concerns | No concerns | No concerns | No concern | Moderate |
| LUR80 vs Placebo | 2 | Low risk | Major concerns | No concerns | No concerns | Major concerns | No concern | Low |

PANSS-N

τ^2^ = 0.000, random-effects design-by-treatment interaction model: p = 0.593

|  | Number of studies | Within-study bias | Across-studies bias | Indirectness | Imprecision | Heterogeneity | Incoherence | Confidence rating |
| --- | --- | --- | --- | --- | --- | --- | --- | --- |
| LUR40 vs LUR80 | 3 | Low risk | Major concerns | No concerns | Major concerns | No concerns | No concern | Low |
| LUR40 vs Placebo | 3 | Low risk | Major concerns | No concerns | No concerns | Major concerns | No concern | Low |
| LUR80 vs Placebo | 2 | Low risk | Major concerns | No concerns | Major concerns | No concerns | No concern | Low |

PANSS-G

τ^2^ = 0.000, random-effects design-by-treatment interaction model: p = 0.229

|  | Number of studies | Within-study bias | Across-studies bias | Indirectness | Imprecision | Heterogeneity | Incoherence | Confidence rating |
| --- | --- | --- | --- | --- | --- | --- | --- | --- |
| LUR40 vs LUR80 | 3 | Low risk | Major concerns | No concerns | No concerns | Major concerns | No concern | Low |
| LUR40 vs Placebo | 3 | Low risk | Major concerns | No concerns | No concerns | No concerns | No concern | Moderate |
| LUR80 vs Placebo | 2 | Low risk | Major concerns | No concerns | Major concerns | No concerns | No concern | Low |

CGI-S

τ^2^ = 0.008, random-effects design-by-treatment interaction model: p = 0.290

|  | Number of studies | Within-study bias | Across-studies bias | Indirectness | Imprecision | Heterogeneity | Incoherence | Confidence rating |
| --- | --- | --- | --- | --- | --- | --- | --- | --- |
| LUR40 vs LUR80 | 2 | Low risk | Major concerns | No concerns | Major concerns | No concerns | No concern | Low |
| LUR40 vs Placebo | 3 | Low risk | Major concerns | No concerns | No concerns | Major concerns | No concern | Low |
| LUR80 vs Placebo | 2 | Low risk | Major concerns | No concerns | No concerns | Major concerns | No concern | Low |

Body weight

τ^2^ = 0.000, random-effects design-by-treatment interaction model: p = 0.991

|  | Number of studies | Within-study bias | Across-studies bias | Indirectness | Imprecision | Heterogeneity | Incoherence | Confidence rating |
| --- | --- | --- | --- | --- | --- | --- | --- | --- |
| LUR40 vs LUR80 | 3 | Low risk | Major concerns | No concerns | Major concerns | No concerns | No concern | Low |
| LUR40 vs Placebo | 3 | Low risk | Major concerns | No concerns | No concerns | Major concerns | No concern | Low |
| LUR80 vs Placebo | 2 | Low risk | Major concerns | No concerns | No concerns | Major concerns | No concern | Low |

Blood triglyceride

τ^2^ = 0.013, random-effects design-by-treatment interaction model: p = 0.026

|  | Number of studies | Within-study bias | Across-studies bias | Indirectness | Imprecision | Heterogeneity | Incoherence | Confidence rating |
| --- | --- | --- | --- | --- | --- | --- | --- | --- |
| LUR40 vs LUR80 | 3 | Low risk | Major concerns | No concerns | Major concerns | No concern | No concern | Low |
| LUR40 vs Placebo | 3 | Low risk | Major concerns | No concerns | Major concerns | No concern | No concern | Low |
| LUR80 vs Placebo | 2 | Low risk | Major concerns | No concerns | Major concerns | No concern | No concern | Low |

Blood total cholesterol

τ^2^ = 0.011, random-effects design-by-treatment interaction model: p = 0.308

|  | Number of studies | Within-study bias | Across-studies bias | Indirectness | Imprecision | Heterogeneity | Incoherence | Confidence rating |
| --- | --- | --- | --- | --- | --- | --- | --- | --- |
| LUR40 vs LUR80 | 3 | Low risk | Major concerns | No concerns | Major concerns | No concern | No concern | Low |
| LUR40 vs Placebo | 3 | Low risk | Major concerns | No concerns | Major concerns | No concern | No concern | Low |
| LUR80 vs Placebo | 2 | Low risk | Major concerns | No concerns | Major concerns | No concern | No concern | Low |

Fasting blood glucose

τ^2^ = 0.000, random-effects design-by-treatment interaction model: p = 0.671

|  | Number of studies | Within-study bias | Across-studies bias | Indirectness | Imprecision | Heterogeneity | Incoherence | Confidence rating |
| --- | --- | --- | --- | --- | --- | --- | --- | --- |
| LUR40 vs LUR80 | 3 | Low risk | Major concerns | No concerns | Major concerns | No concern | No concern | Low |
| LUR40 vs Placebo | 3 | Low risk | Major concerns | No concerns | Major concerns | No concern | No concern | Low |
| LUR80 vs Placebo | 2 | Low risk | Major concerns | No concerns | Major concerns | No concern | No concern | Low |

Blood HbA1c

τ^2^ = 0.008, random-effects design-by-treatment interaction model: p = 0.064

|  | Number of studies | Within-study bias | Across-studies bias | Indirectness | Imprecision | Heterogeneity | Incoherence | Confidence rating |
| --- | --- | --- | --- | --- | --- | --- | --- | --- |
| LUR40 vs LUR80 | 3 | Low risk | Major concerns | No concerns | Major concerns | No concern | No concern | Low |
| LUR40 vs Placebo | 3 | Low risk | Major concerns | No concerns | Major concerns | No concern | Major concerns | Very low |
| LUR80 vs Placebo | 2 | Low risk | Major concerns | No concerns | Major concerns | No concern | No concern | Low |

Blood prolactin

τ^2^ = 0.006, random-effects design-by-treatment interaction model: p = 0.101

|  | Number of studies | Within-study bias | Across-studies bias | Indirectness | Imprecision | Heterogeneity | Incoherence | Confidence rating |
| --- | --- | --- | --- | --- | --- | --- | --- | --- |
| LUR40 vs LUR80 | 3 | Low risk | Major concerns | No concerns | Major concerns | No concern | No concern | Low |
| LUR40 vs Placebo | 3 | Low risk | Major concerns | No concerns | Major concerns | No concern | No concern | Low |
| LUR80 vs Placebo | 2 | Low risk | Major concerns | No concerns | Major concerns | No concern | No concern | Low |

DIEPSS

τ^2^ = 0.002, random-effects design-by-treatment interaction model: p = 0.136

|  | Number of studies | Within-study bias | Across-studies bias | Indirectness | Imprecision | Heterogeneity | Incoherence | Confidence rating |
| --- | --- | --- | --- | --- | --- | --- | --- | --- |
| LUR40 vs LUR80 | 3 | Low risk | Major concerns | No concerns | No concern | Major concerns | No concern | Low |
| LUR40 vs Placebo | 3 | Low risk | Major concerns | No concerns | Major concerns | No concern | No concern | Low |
| LUR80 vs Placebo | 2 | Low risk | Major concerns | No concerns | No concern | No concern | No concern | Moderate |

Response rate

τ^2^ = 0.006, random-effects design-by-treatment interaction model: p = 0.027

|  | Number of studies | Within-study bias | Across-studies bias | Indirectness | Imprecision | Heterogeneity | Incoherence | Confidence rating |
| --- | --- | --- | --- | --- | --- | --- | --- | --- |
| LUR40 vs LUR80 | 2 | Low risk | Major concerns | No concerns | Major concerns | No concerns | No concern | Low |
| LUR40 vs Placebo | 3 | Low risk | Major concerns | No concerns | No concerns | Major concerns | Major concerns | Very low |
| LUR80 vs Placebo | 2 | Low risk | Major concerns | No concerns | Major concerns | No concerns | Major concerns | Very low |

All-cause discontinuation

τ^2^ = 0.002, random-effects design-by-treatment interaction model: p = 0.197

|  | Number of studies | Within-study bias | Across-studies bias | Indirectness | Imprecision | Heterogeneity | Incoherence | Confidence rating |
| --- | --- | --- | --- | --- | --- | --- | --- | --- |
| LUR40 vs LUR80 | 3 | Low risk | Major concerns | No concerns | Major concerns | No concerns | No concern | Low |
| LUR40 vs Placebo | 3 | Low risk | Major concerns | No concerns | Major concerns | No concerns | Major concerns | Very low |
| LUR80 vs Placebo | 2 | Low risk | Major concerns | No concerns | Major concerns | No concerns | Major concerns | Very low |

Discontinuation due to adverse events

τ^2^ = 0.045, random-effects design-by-treatment interaction model: p = 0.091

|  | Number of studies | Within-study bias | Across-studies bias | Indirectness | Imprecision | Heterogeneity | Incoherence | Confidence rating |
| --- | --- | --- | --- | --- | --- | --- | --- | --- |
| LUR40 vs LUR80 | 3 | Low risk | Major concerns | No concerns | Major concerns | No concerns | No concern | Low |
| LUR40 vs Placebo | 3 | Low risk | Major concerns | No concerns | Major concerns | No concerns | Major concerns | Very low |
| LUR80 vs Placebo | 2 | Low risk | Major concerns | No concerns | Major concerns | No concerns | No concern | Low |

At least one adverse event

τ^2^ = 0.000, random-effects design-by-treatment interaction model: p = 0.229

|  | Number of studies | Within-study bias | Across-studies bias | Indirectness | Imprecision | Heterogeneity | Incoherence | Confidence rating |
| --- | --- | --- | --- | --- | --- | --- | --- | --- |
| LUR40 vs LUR80 | 3 | Low risk | Major concerns | No concerns | Major concerns | No concerns | No concern | Low |
| LUR40 vs Placebo | 3 | Low risk | Major concerns | No concerns | Major concerns | No concerns | No concern | Low |
| LUR80 vs Placebo | 2 | Low risk | Major concerns | No concerns | Major concerns | No concerns | No concern | Low |

Akathisia

τ^2^ = 0.000, random-effects design-by-treatment interaction model: p = 0.810

|  | Number of studies | Within-study bias | Across-studies bias | Indirectness | Imprecision | Heterogeneity | Incoherence | Confidence rating |
| --- | --- | --- | --- | --- | --- | --- | --- | --- |
| LUR40 vs LUR80 | 3 | Low risk | Major concerns | No concerns | Major concerns | No concerns | No concern | Low |
| LUR40 vs Placebo | 3 | Low risk | Major concerns | No concerns | No concerns | No concerns | No concern | Moderate |
| LUR80 vs Placebo | 2 | Low risk | Major concerns | No concerns | No concerns | No concerns | No concern | Moderate |

Anxiety

τ^2^ = 0.303, random-effects design-by-treatment interaction model: p = 0.864

|  | Number of studies | Within-study bias | Across-studies bias | Indirectness | Imprecision | Heterogeneity | Incoherence | Confidence rating |
| --- | --- | --- | --- | --- | --- | --- | --- | --- |
| LUR40 vs LUR80 | 3 | Low risk | Major concerns | No concerns | Major concerns | No concerns | No concern | Low |
| LUR40 vs Placebo | 3 | Low risk | Major concerns | No concerns | Major concerns | Major concerns | No concern | Very low |
| LUR80 vs Placebo | 2 | Low risk | Major concerns | No concerns | Major concerns | No concerns | No concern | Low |

Constipation

τ^2^ = 0.036, random-effects design-by-treatment interaction model: p = 0.371

|  | Number of studies | Within-study bias | Across-studies bias | Indirectness | Imprecision | Heterogeneity | Incoherence | Confidence rating |
| --- | --- | --- | --- | --- | --- | --- | --- | --- |
| LUR40 vs LUR80 | 3 | Low risk | Major concerns | No concerns | Major concerns | No concerns | No concern | Low |
| LUR40 vs Placebo | 3 | Low risk | Major concerns | No concerns | Major concerns | No concerns | No concern | Low |
| LUR80 vs Placebo | 2 | Low risk | Major concerns | No concerns | Major concerns | No concerns | No concern | Low |

Diarrhea

τ^2^ = 0.000, random-effects design-by-treatment interaction model: p = 0.761

|  | Number of studies | Within-study bias | Across-studies bias | Indirectness | Imprecision | Heterogeneity | Incoherence | Confidence rating |
| --- | --- | --- | --- | --- | --- | --- | --- | --- |
| LUR40 vs LUR80 | 3 | Low risk | Major concerns | No concerns | Major concerns | No concerns | No concern | Low |
| LUR40 vs Placebo | 3 | Low risk | Major concerns | No concerns | Major concerns | No concerns | No concern | Low |
| LUR80 vs Placebo | 2 | Low risk | Major concerns | No concerns | Major concerns | No concerns | No concern | Low |

Dizziness

τ^2^ = 0.000, random-effects design-by-treatment interaction model: p = 0.953

|  | Number of studies | Within-study bias | Across-studies bias | Indirectness | Imprecision | Heterogeneity | Incoherence | Confidence rating |
| --- | --- | --- | --- | --- | --- | --- | --- | --- |
| LUR40 vs LUR80 | 3 | Low risk | Major concerns | No concerns | Major concerns | No concerns | No concern | Low |
| LUR40 vs Placebo | 3 | Low risk | Major concerns | No concerns | Major concerns | No concerns | No concern | Low |
| LUR80 vs Placebo | 2 | Low risk | Major concerns | No concerns | Major concerns | No concerns | No concern | Low |

Dry mouth

τ^2^ = 0.000, random-effects design-by-treatment interaction model: p = 0.894

|  | Number of studies | Within-study bias | Across-studies bias | Indirectness | Imprecision | Heterogeneity | Incoherence | Confidence rating |
| --- | --- | --- | --- | --- | --- | --- | --- | --- |
| LUR40 vs LUR80 | 3 | Low risk | Major concerns | No concerns | Major concerns | No concerns | No concern | Low |
| LUR40 vs Placebo | 3 | Low risk | Major concerns | No concerns | Major concerns | No concerns | No concern | Low |
| LUR80 vs Placebo | 2 | Low risk | Major concerns | No concerns | Major concerns | No concerns | No concern | Low |

Dystonia

τ^2^ = 0.000, random-effects design-by-treatment interaction model: p = 0.934

|  | Number of studies | Within-study bias | Across-studies bias | Indirectness | Imprecision | Heterogeneity | Incoherence | Confidence rating |
| --- | --- | --- | --- | --- | --- | --- | --- | --- |
| LUR40 vs LUR80 | 3 | Low risk | Major concerns | No concerns | No concerns | No concerns | No concern | Moderate |
| LUR40 vs Placebo | 3 | Low risk | Major concerns | No concerns | No concerns | No concerns | No concern | Moderate |
| LUR80 vs Placebo | 2 | Low risk | Major concerns | No concerns | Major concerns | Major concerns | No concern | Very low |

Headache

τ^2^ = 0.000, random-effects design-by-treatment interaction model: p = 0.912

|  | Number of studies | Within-study bias | Across-studies bias | Indirectness | Imprecision | Heterogeneity | Incoherence | Confidence rating |
| --- | --- | --- | --- | --- | --- | --- | --- | --- |
| LUR40 vs LUR80 | 3 | Low risk | Major concerns | No concerns | Major concerns | No concerns | No concern | Low |
| LUR40 vs Placebo | 3 | Low risk | Major concerns | No concerns | Major concerns | No concerns | No concern | Low |
| LUR80 vs Placebo | 2 | Low risk | Major concerns | No concerns | Major concerns | Major concerns | No concern | Very low |

Insomnia

τ^2^ = 0.051, random-effects design-by-treatment interaction model: p = 0.094

|  | Number of studies | Within-study bias | Across-studies bias | Indirectness | Imprecision | Heterogeneity | Incoherence | Confidence rating |
| --- | --- | --- | --- | --- | --- | --- | --- | --- |
| LUR40 vs LUR80 | 3 | Low risk | Major concerns | No concerns | Major concerns | No concerns | No concern | Low |
| LUR40 vs Placebo | 3 | Low risk | Major concerns | No concerns | Major concerns | No concerns | No concern | Low |
| LUR80 vs Placebo | 2 | Low risk | Major concerns | No concerns | Major concerns | No concerns | No concern | Low |

Muscle rigidity

τ^2^ = 0.000, random-effects design-by-treatment interaction model: p = 0.707

|  | Number of studies | Within-study bias | Across-studies bias | Indirectness | Imprecision | Heterogeneity | Incoherence | Confidence rating |
| --- | --- | --- | --- | --- | --- | --- | --- | --- |
| LUR40 vs LUR80 | 3 | Low risk | Major concerns | No concerns | Major concerns | No concerns | No concern | Low |
| LUR40 vs Placebo | 3 | Low risk | Major concerns | No concerns | Major concerns | No concerns | No concern | Low |
| LUR80 vs Placebo | 2 | Low risk | Major concerns | No concerns | Major concerns | No concerns | No concern | Low |

Nausea

τ^2^ = 0.036, random-effects design-by-treatment interaction model: p = 0.188

|  | Number of studies | Within-study bias | Across-studies bias | Indirectness | Imprecision | Heterogeneity | Incoherence | Confidence rating |
| --- | --- | --- | --- | --- | --- | --- | --- | --- |
| LUR40 vs LUR80 | 3 | Low risk | Major concerns | No concerns | Major concerns | No concerns | No concern | Low |
| LUR40 vs Placebo | 3 | Low risk | Major concerns | No concerns | Major concerns | No concerns | No concern | Low |
| LUR80 vs Placebo | 2 | Low risk | Major concerns | No concerns | Major concerns | No concerns | No concern | Low |

Rash

τ^2^ = 0.000, random-effects design-by-treatment interaction model: p = 0.980

|  | Number of studies | Within-study bias | Across-studies bias | Indirectness | Imprecision | Heterogeneity | Incoherence | Confidence rating |
| --- | --- | --- | --- | --- | --- | --- | --- | --- |
| LUR40 vs LUR80 | 3 | Low risk | Major concerns | No concerns | Major concerns | No concerns | No concern | Low |
| LUR40 vs Placebo | 3 | Low risk | Major concerns | No concerns | Major concerns | No concerns | No concern | Low |
| LUR80 vs Placebo | 2 | Low risk | Major concerns | No concerns | Major concerns | No concerns | No concern | Low |

Schizophrenia

τ^2^ = 0.000, random-effects design-by-treatment interaction model: p = 0.944

|  | Number of studies | Within-study bias | Across-studies bias | Indirectness | Imprecision | Heterogeneity | Incoherence | Confidence rating |
| --- | --- | --- | --- | --- | --- | --- | --- | --- |
| LUR40 vs LUR80 | 3 | Low risk | Major concerns | No concerns | Major concerns | No concerns | No concern | Low |
| LUR40 vs Placebo | 3 | Low risk | Major concerns | No concerns | No concerns | Major concerns | No concern | Low |
| LUR80 vs Placebo | 2 | Low risk | Major concerns | No concerns | Major concerns | No concerns | No concern | Low |

Serious adverse event

τ^2^ = 0.000, random-effects design-by-treatment interaction model: p = 0.751

|  | Number of studies | Within-study bias | Across-studies bias | Indirectness | Imprecision | Heterogeneity | Incoherence | Confidence rating |
| --- | --- | --- | --- | --- | --- | --- | --- | --- |
| LUR40 vs LUR80 | 3 | Low risk | Major concerns | No concerns | Major concerns | No concerns | No concern | Low |
| LUR40 vs Placebo | 3 | Low risk | Major concerns | No concerns | Major concerns | No concerns | No concern | Low |
| LUR80 vs Placebo | 2 | Low risk | Major concerns | No concerns | Major concerns | No concerns | No concern | Low |

Somnolence

τ^2^ = 0.000, random-effects design-by-treatment interaction model: p = 0.651

|  | Number of studies | Within-study bias | Across-studies bias | Indirectness | Imprecision | Heterogeneity | Incoherence | Confidence rating |
| --- | --- | --- | --- | --- | --- | --- | --- | --- |
| LUR40 vs LUR80 | 3 | Low risk | Major concerns | No concerns | Major concerns | No concerns | No concern | Low |
| LUR40 vs Placebo | 3 | Low risk | Major concerns | No concerns | No concerns | Major concerns | No concern | Low |
| LUR80 vs Placebo | 2 | Low risk | Major concerns | No concerns | No concerns | Major concerns | No concern | Low |

Tremor

τ^2^ = 0.000, random-effects design-by-treatment interaction model: p = 0.598

|  | Number of studies | Within-study bias | Across-studies bias | Indirectness | Imprecision | Heterogeneity | Incoherence | Confidence rating |
| --- | --- | --- | --- | --- | --- | --- | --- | --- |
| LUR40 vs LUR80 | 3 | Low risk | Major concerns | No concerns | Major concerns | No concerns | No concern | Low |
| LUR40 vs Placebo | 3 | Low risk | Major concerns | No concerns | Major concerns | No concerns | No concern | Low |
| LUR80 vs Placebo | 2 | Low risk | Major concerns | No concerns | Major concerns | No concerns | No concern | Low |

Use of sleeping pills

τ^2^ = 0.000, random-effects design-by-treatment interaction model: p = 0.450

|  | Number of studies | Within-study bias | Across-studies bias | Indirectness | Imprecision | Heterogeneity | Incoherence | Confidence rating |
| --- | --- | --- | --- | --- | --- | --- | --- | --- |
| LUR40 vs LUR80 | 2 | Low risk | Major concerns | No concerns | Major concerns | No concerns | No concern | Low |
| LUR40 vs Placebo | 3 | Low risk | Major concerns | No concerns | Major concerns | No concerns | No concern | Low |
| LUR80 vs Placebo | 2 | Low risk | Major concerns | No concerns | Major concerns | No concerns | No concern | Low |

Use of anxiolytic

τ^2^ = 0.000, random-effects design-by-treatment interaction model: p = 0.130

|  | Number of studies | Within-study bias | Across-studies bias | Indirectness | Imprecision | Heterogeneity | Incoherence | Confidence rating |
| --- | --- | --- | --- | --- | --- | --- | --- | --- |
| LUR40 vs LUR80 | 2 | Low risk | Major concerns | No concerns | Major concerns | No concerns | No concern | Low |
| LUR40 vs Placebo | 3 | Low risk | Major concerns | No concerns | Major concerns | No concerns | No concern | Low |
| LUR80 vs Placebo | 2 | Low risk | Major concerns | No concerns | Major concerns | No concerns | No concern | Low |

Use of Anticholinergic drugs

τ^2^ = 0.086, random-effects design-by-treatment interaction model: p = 0.128

|  | Number of studies | Within-study bias | Across-studies bias | Indirectness | Imprecision | Heterogeneity | Incoherence | Confidence rating |
| --- | --- | --- | --- | --- | --- | --- | --- | --- |
| LUR40 vs LUR80 | 3 | Low risk | Major concerns | No concerns | Major concerns | No concerns | No concern | Low |
| LUR40 vs Placebo | 3 | Low risk | Major concerns | No concerns | Major concerns | No concerns | No concern | Low |
| LUR80 vs Placebo | 2 | Low risk | Major concerns | No concerns | Major concerns | No concerns | No concern | Low |

Vomiting

τ^2^ = 0.000, random-effects design-by-treatment interaction model: p = 0.636

|  | Number of studies | Within-study bias | Across-studies bias | Indirectness | Imprecision | Heterogeneity | Incoherence | Confidence rating |
| --- | --- | --- | --- | --- | --- | --- | --- | --- |
| LUR40 vs LUR80 | 3 | Low risk | Major concerns | No concerns | Major concerns | No concerns | No concern | Low |
| LUR40 vs Placebo | 3 | Low risk | Major concerns | No concerns | Major concerns | No concerns | No concern | Low |
| LUR80 vs Placebo | 2 | Low risk | Major concerns | No concerns | Major concerns | No concerns | No concern | Low |

Weight gain (> 7%)

τ^2^ = 0.000, random-effects design-by-treatment interaction model: p = 0.553

|  | Number of studies | Within-study bias | Across-studies bias | Indirectness | Imprecision | Heterogeneity | Incoherence | Confidence rating |
| --- | --- | --- | --- | --- | --- | --- | --- | --- |
| LUR40 vs LUR80 | 2 | Low risk | Major concerns | No concerns | Major concerns | No concerns | No concern | Low |
| LUR40 vs Placebo | 3 | Low risk | Major concerns | No concerns | No concerns | No concerns | No concern | Moderate |
| LUR80 vs Placebo | 2 | Low risk | Major concerns | No concerns | Major concerns | No concerns | No concern | Low |

Weight loss (> 7%)

τ^2^ = 0.000, random-effects design-by-treatment interaction model: p = 0.874

|  | Number of studies | Within-study bias | Across-studies bias | Indirectness | Imprecision | Heterogeneity | Incoherence | Confidence rating |
| --- | --- | --- | --- | --- | --- | --- | --- | --- |
| LUR40 vs LUR80 | 2 | Low risk | Major concerns | No concerns | Major concerns | No concerns | No concern | Low |
| LUR40 vs Placebo | 3 | Low risk | Major concerns | No concerns | Major concerns | No concerns | No concern | Low |
| LUR80 vs Placebo | 2 | Low risk | Major concerns | No concerns | No concerns | No concerns | No concern | Moderate |

QTcF interval 450msec

τ^2^ = 0.011, random-effects design-by-treatment interaction model: p = 0.413

|  | Number of studies | Within-study bias | Across-studies bias | Indirectness | Imprecision | Heterogeneity | Incoherence | Confidence rating |
| --- | --- | --- | --- | --- | --- | --- | --- | --- |
| LUR40 vs LUR80 | 2 | Low risk | Major concerns | No concerns | Major concerns | No concerns | No concern | Low |
| LUR40 vs Placebo | 3 | Low risk | Major concerns | No concerns | Major concerns | No concerns | No concern | Low |
| LUR80 vs Placebo | 2 | Low risk | Major concerns | No concerns | Major concerns | No concerns | No concern | Low |
